# Enhanced Hydrogen Detection in ppb-Level by Electrospun SnO_2_-Loaded ZnO Nanofibers

**DOI:** 10.3390/s19030726

**Published:** 2019-02-11

**Authors:** Jae-Hyoung Lee, Jin-Young Kim, Jae-Hun Kim, Sang Sub Kim

**Affiliations:** Department of Materials Science and Engineering, Inha University, Incheon 22212, Korea; jhlee5321@naver.com (J.-H.L.); piadote@naver.com (J.-Y.K.); kjhhb5331@gmail.com (J.-H.K.)

**Keywords:** SnO_2_, electrospinning, nanofiber, sensing mechanism, hydrogen gas

## Abstract

High-performance hydrogen sensors are important in many industries to effectively address safety concerns related to the production, delivering, storage and use of H_2_ gas. Herein, we present a highly sensitive hydrogen gas sensor based on SnO_2_-loaded ZnO nanofibers (NFs). The xSnO_2_-loaded (x = 0.05, 0.1 and 0.15) ZnO NFs were fabricated using an electrospinning technique followed by calcination at high temperature. Microscopic analyses demonstrated the formation of NFs with expected morphology and chemical composition. Hydrogen sensing studies were performed at various temperatures and the optimal working temperature was selected as 300 °C. The optimal gas sensor (0.1 SnO_2_ loaded ZnO NFs) not only showed a high response to 50 ppb hydrogen gas, but also showed an excellent selectivity to hydrogen gas. The excellent performance of the gas sensor to hydrogen gas was mainly related to the formation of SnO_2_-ZnO heterojunctions and the metallization effect of ZnO.

## 1. Introduction

Hydrogen gas (H_2_) is a highly clean and renewable source of energy [1]. Nevertheless, it is flammable and explosive in the concentration ranges of 4–75 vol.% in air [2]. Due to the small size of hydrogen gas during its transportation, storage and usages, leakage of hydrogen is likely and can cause catastrophic damage [3]. Unfortunately, hydrogen is a colorless, odorless and tasteless gas and it is impossible to be detected by human senses [4]. Therefore, detection of hydrogen gas by high-performance electronic devices is very important for further industrial use of this gas.

So far, many hydrogen gas sensors, such as surface acoustic wave [5], optical [6], gasochromic [7], thermoelectric [8] and metal oxide-based sensors [9] have been developed. Among them, metal oxide-based gas sensors are widely used for the detection of different toxic gases and volatile organic compounds, thanks to their low price, simple fabrication, good sensitivity and high stability [10,11,12]. To date, many types of metal oxides, including binary metal oxides [13] and ternary metal oxides [14], have been reported for detection of hydrogen gas. Among them, ZnO-based gas sensors are highly attentive due to having high sensitivity, high stability, easy synthesis methods and a low-cost [15]. In particular, one-dimensional (1D) morphologies of ZnO, like nanorods [16], nanowires [17], and nanofibers (NFs) [18], have gained special attention, mostly due to their simple synthesis methods and larger surface area relative to thin and thick film counterparts. ZnO NFs are among the simplest morphology of ZnO. ZnO NFs can be easily synthesized by the electrospinning technique, which is a facile and low-cost synthesis method with possibility of large-scale mass production for industrialization [19,20,21]. Accordingly, many researchers have reported gas sensors using ZnO NFs [22,23].

Doping can increase the structural defect within the ZnO, acting as potential adsorption sites for target gases [24]. Accordingly, doping with different dopants is also a promising strategy to improve the gas sensing characteristics of 1D ZnO gas sensors [25,26,27]. Another promising strategy is loading of n-type [28] or p-type [29] metal oxides in the matrix of the ZnO nanomaterials. Loaded metal oxides can form heterojunctions with ZnO, acting as a strong source of resistance modulation in the gas sensor.

So far, less attention has been paid to SnO_2_ loading on ZnO NFs. Accordingly, in this research, we have fabricated xSnO_2_-loaded (x = 0.05, 0.1 and 0.15) ZnO NFs for hydrogen gas sensing investigations. They were synthesized by the electrospinning method, followed by calcination. The hydrogen gas sensing properties showed that the gas sensor with 0.1 SnO_2_ loading had the best sensing properties at 300 °C. The sensing mechanism was explained due to the formation of n-n SnO_2_-ZnO heterojunctions and the metallization effect of ZnO in the presence of hydrogen gas.

## 2. Experimental Procedure

### 2.1. Synthesis of SnO_2_-Loaded ZnO NFs

**Figure 1:**
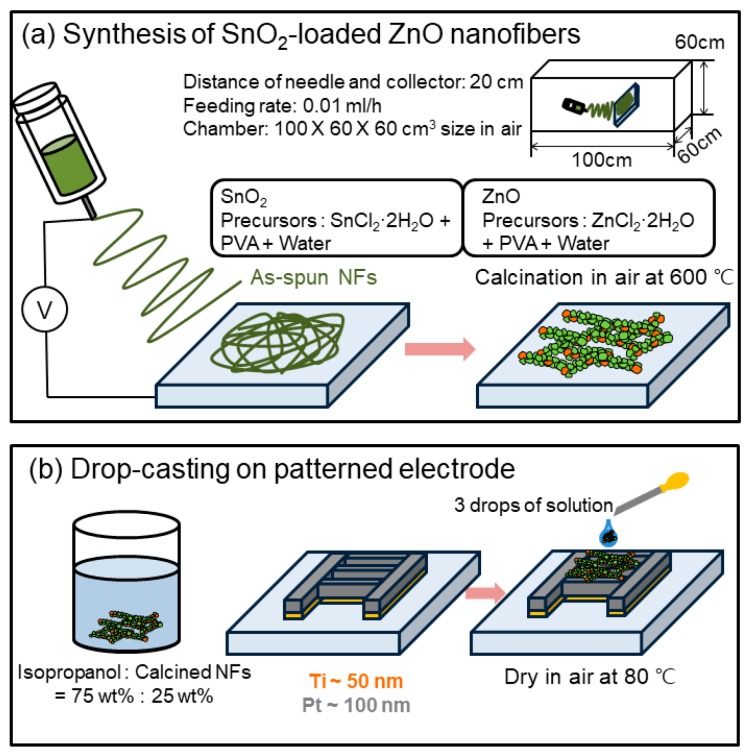
Illustrations of (**a**) the electrospinning method for the preparation of xSnO_2_-loaded (x = 0.05, 0.1 and 0.15) ZnO nanofibers (NFs) and (**b**) drop casting of NFs onto the patterned electrode of the sensing substrate.

For synthesis of xSnO_2_-loaded (x = 0.05, 0.1 and 0.15) ZnO NFs: Polyvinyl alcohol (PVA, MW = 80,000), zinc chloride dihydrate (ZnCl_2_·2H_2_O) and tin (II) chloride dihydrate (SnCl_2_·2H_2_O) were provided by Sigma-Aldrich. First, the PVA was dissolved in deionized (DI) water and stirred at 80 °C for 3 h to prepare a 10 wt.% PVA solution. Then, 1 g of ZnCl_2_·2H_2_O and desired amounts (x = 0.05, 0.1 and 0.15) of tin precursor were added drop-wise (0.05 mL/h) to the above solution and vigorously stirred for 12 h at 80 °C. Then final solutions with an approximate viscosity of 280 mPa·s were prepared. The electrospinning solution was loaded into a syringe with a metallic needle. A large positive voltage (+15 kV) and large negative voltage (−10 kV) were applied to the needle and Al collector, respectively. During the process, both the distance between the tip of the needle and collector (20 cm) and the feed rate (0.01 mL/h) were fixed. It should be noted that the electrospinning of NFs was performed in a chamber with dimensions of 100 × 60 × 60 cm^3^, which was specially designed for the electrospinning machine. After synthesis of the SnO_2_-loaded ZnO NFs, they were annealed at 600 °C for 2 h to enhance the crystallinity and remove the remaining organic species and water. Figure 1a shows the schematic of electrospinning for the preparation of SnO_2_-loaded ZnO NFs.

### 2.2. Device for Material Characterization

The morphology of the synthesized NFs were obtained by using field-emission scanning electron microscopy (FE-SEM, Hitachi, S-4200, Tokyo, Japan) and transmission electron microscopy (TEM, JEOL, Ltd., JEM-3010, Tokyo, Japan) incorporated with energy-dispersive X-ray spectroscopy (EDS, JEOL, Ltd., JEM-3010, Tokyo, Japan) for chemical analysis of the NFs.

### 2.3. Gas Sensing Test

Details of the sensing test methods are presented in our previous papers [18,30]. First, Ti (∼50 nm thick) and Pt (∼100 nm thick) bilayer electrodes were sputter-deposited onto SiO_2_-coated Si substrates and then NFs were drop casted (3 droplets, followed by drying at 80 °C) onto the substrate (Figure 1b). Second, for the sensing tests, the sensors were put into a tube furnace, equipped with a gas chamber that can control the temperature. Gas concentrations were controlled exactly, by varying the ratios of the desired gases to dry air using mass flow controllers. After recording the resistance in air (R_a_) and the resistance in the presence of the target gas (R_g_) by means of a Keithly source meter, the sensor response (R) was calculated as R = R_a_/R_g_ (for H_2_ and CO gases) and R = R_g_/R_a_ (for NO_2_ gas) [31,32,33,34,35,36,37,38,39]. The response time and the recovery time were defined as the time needed for the resistance to reach 90% of its final value upon exposure to target gas and air, respectively [40].

## 3. Results and Discussion

### 3.1. Morphological and Microstructural Study

**Figure 2:**
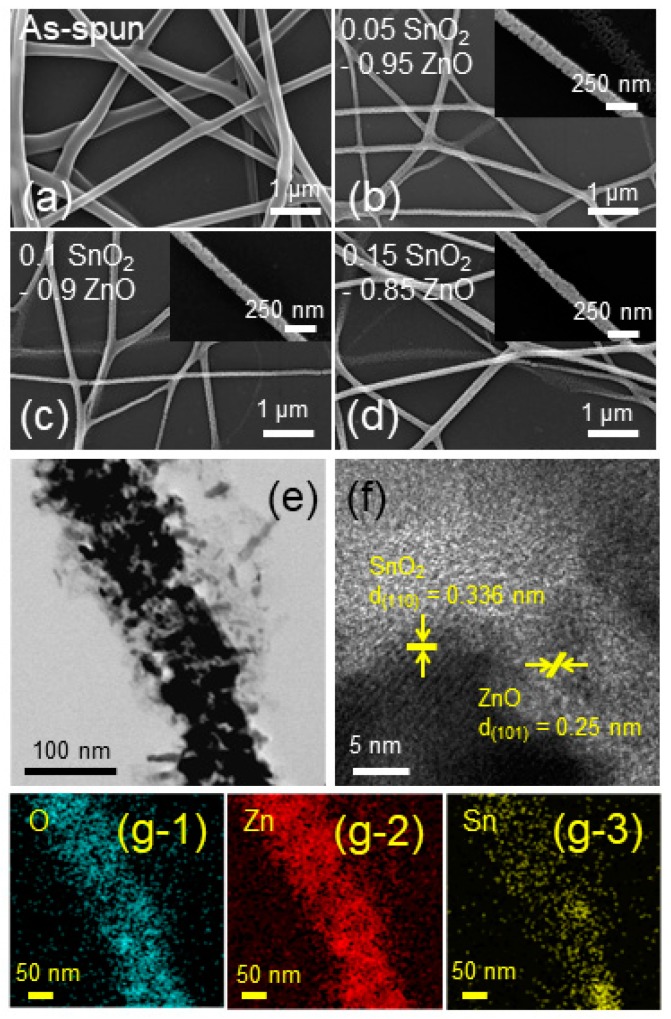
Field-emission scanning electron microscopy (FE-SEM) micrographs of (**a**) as-spun 0.1 SnO_2_-loaded ZnO NFs and calcined, (**b**) 0.05 SnO_2_-loaded ZnO NFs, (**c**) 0.1 SnO_2_-loaded ZnO NFs, and (**d**) 0.15 SnO_2_-loaded ZnO NFs. Insets in (**b**–**d**) show corresponding higher magnification FE-SEM images. (**e**) Low-magnification TEM image and (**f**) high-resolution TEM image of 0.1 SnO_2_-loaded ZnO NFs. (**g-1**), (**g-2**) and (**g-3**) energy-dispersive X-ray spectroscopy (EDS) O, Zn and Sn elemental mapping of 0.1 SnO_2_-loaded ZnO NFs taken from (**e**), respectively.

An FE-SEM micrograph of the as-electrospun 0.1 SnO_2_-loaded ZnO NFs is provided in Figure 2a. As shown, long and continuous ZnO NFs have been successfully synthesized. However, since they were not yet calcined, their surface morphology was quite smooth. The FE-SEM micrographs of the calcined SnO_2_-loaded ZnO NFs are shown in Figure 2b,c for 0.05, 0.1 and 0.15 SnO_2_-loaded ZnO NFs, respectively. The calcined individual NFs were still long and continuous, but showed a grainy morphology, which indicated evaporation of water and the removal of organic species from the as-electrospun samples. Apparently after calcination, the diameter of the calcined ZnO NFs decreased relative to the as-electrospun NFs. The insets show higher magnification FE-SEM images, which demonstrated the presence of nano-sized grains. 

**Figure 3:**
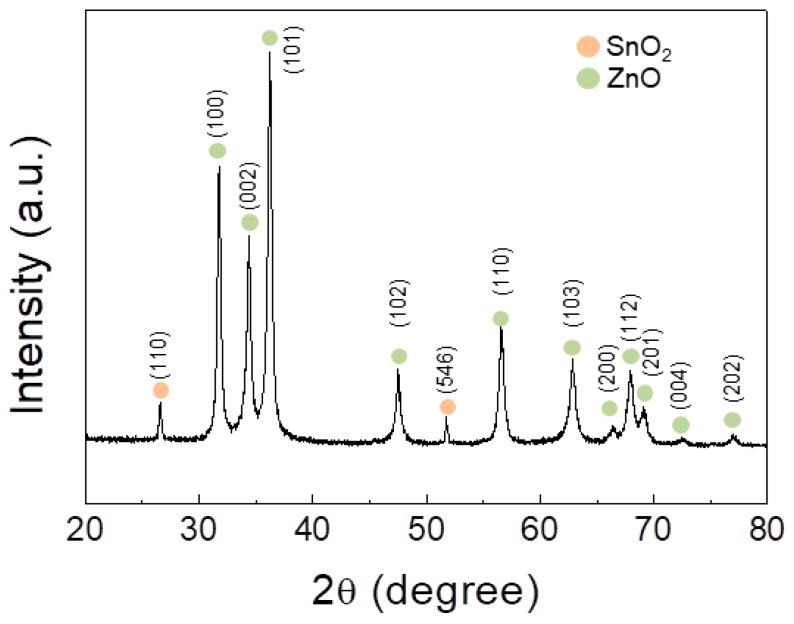
XRD pattern of the 0.1 SnO_2_-loaded ZnO NFs.

TEM observation was performed to further characterize the microstructure of the NFs. A representative TEM image taken from the 0.1 SnO_2_-loaded ZnO NFs is presented in Figure 2e. This clearly demonstrated the formation of a NF of an approximate diameter of 100 nm with rugged surface morphology. To gain an insight into the crystallinity of calcined 0.1 SnO_2_-loaded ZnO NFs, a high-resolution TEM micrograph was obtained as shown in Figure 2f. Parallel fringes with spacings of 0.336 nm and 0.250 nm can be attributed to the crystalline planes of (110) of SnO_2_ and (101) of ZnO, respectively [41,42]. This indicated the formation of crystalline ZnO and SnO_2_ phases after calcination. EDS mapping analysis results, shown in Figure 2(g-1,g-2,g-3) taken from Figure 2e, demonstrated the existence of O, Zn and Sn elements in the NF. 

Figure 3 shows a representative XRD pattern of the 0.1 SnO_2_-loaded ZnO NFs. The obtained pattern matches well with both ZnO and SnO_2_ phases. It shows the existence of both hexagonal ZnO (JCPDS Card No. 88-0511) and tetragonal SnO_2_ (JCPDS Card No. 88-0287) crystal structures. 

### 3.2. Gas Sensing Study

**Figure 4:**
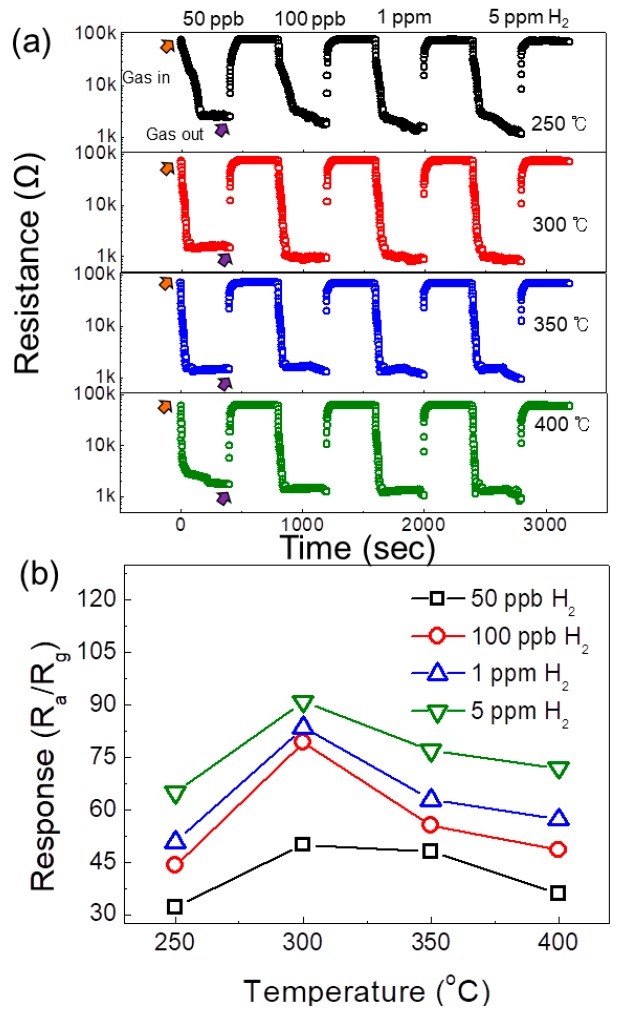
(**a**) Dynamic response curves of 0.1 SnO_2_-loaded ZnO NFs to 50 ppb, 100 ppb, 1 ppm and 5 ppm H_2_ gas at different temperatures. (**b**) Calculated hydrogen response as a function of operating temperature.

Adsorption phenomena are strongly dependent on the sensing temperature of the gas sensor and generally, there is an optimal sensing temperature, where the maximum response occurs. As a first step, a 0.15 SnO_2_-loaded ZnO NFs gas sensor was exposed to different concentrations of hydrogen gas at temperature range of 250–400 °C and corresponding transient resistance plots are shown in Figure 4a. As shown, upon injection of hydrogen gas, the resistance of the gas sensor decreased, revealing a n-type nature of gas sensor, which originated from the n-type semiconducting characteristics of ZnO and SnO_2_ materials. To see the response of the gas sensor to various concentrations of hydrogen gas, the corresponding calibration plots are depicted in Figure 4b. At 300 °C for all concentrations of hydrogen gas, a maximum response was observed and the gas sensor even could detect as low as 50 ppb hydrogen gas. At the optimal sensing temperature (300 °C), the responses of the gas sensor to 50 ppb, 100 ppb, 1 ppm, and 5 ppm were 50.1, 79.4, 83.62, and 91, respectively. This demonstrated a very high response of the gas sensor to hydrogen gas. 

**Figure 5:**
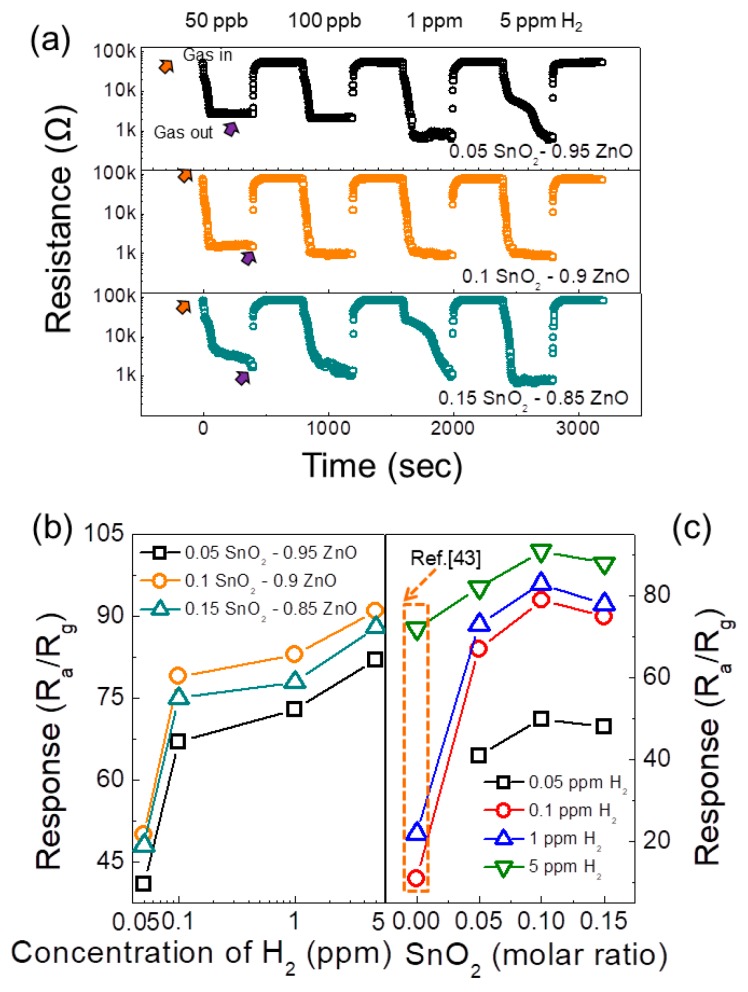
(**a**) Dynamic response curves of SnO_2_-loaded ZnO NFs gas sensors to 50 ppb, 100 ppb, 1 ppm and 5 ppm H_2_ gas at 300 °C. (**b**) Calculated hydrogen response as a function of hydrogen concentration. (**c**) Calculated hydrogen response as a function of SnO_2_ loading amount.

In the next step, different contents of SnO_2_-loaded gas sensors were exposed to various concentrations of hydrogen gas at 300 °C and corresponding transient curves are provided in Figure 5a. The sensor responses are summarized in Figure 5b,c which shows the following order in the sensor response to hydrogen gas: 0.1 > 0.15 > 0.05 SnO_2_-loaded ZnO NFs gas sensor. For example, for 50 ppb hydrogen gas, the responses of 0.05, 0.1 and 0.15 molar ratio of SnO_2_-loaded ZnO gas sensors were 41, 48 and 50.1, respectively. Accordingly, the 0.1 SnO_2_-loaded ZnO NFs gas sensor was selected as the optimal gas sensor. The responses of all gas were higher than that of the pristine ZnO NFs gas sensor [43].

**Figure 6:**
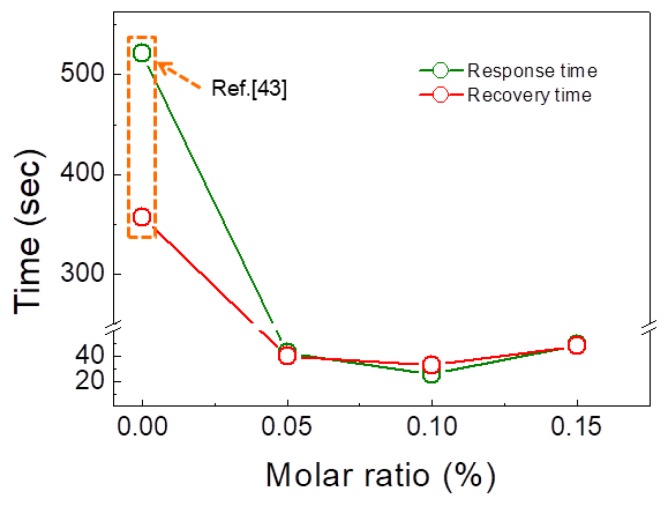
Response and recovery times of xSnO_2_ (x = 0, 0.05, 0.1 and 0.15) loaded ZnO NFs to 5 ppm H_2_ gas at 300 °C.

Since selectivity of the gas sensor is of importance for practical usages, the optimized gas sensor was exposed to different concentrations of NO_2_ and CO gases, which are typical oxidizing and reducing gases, respectively. Figure 6 shows the response time and recovery time of xSnO_2_ (x = 0, 0.05, 0.1 and 0.15) loaded ZnO NF gas sensors to 5 ppm H_2_ gas at 300 °C. The response and recovery times significantly decreased with increasing “x”. Also the 0.1 SnO_2_-loaded ZnO NFs gas sensor showed the shortest response and recovery times, compared to the sensors of other compositions.

**Figure 7:**
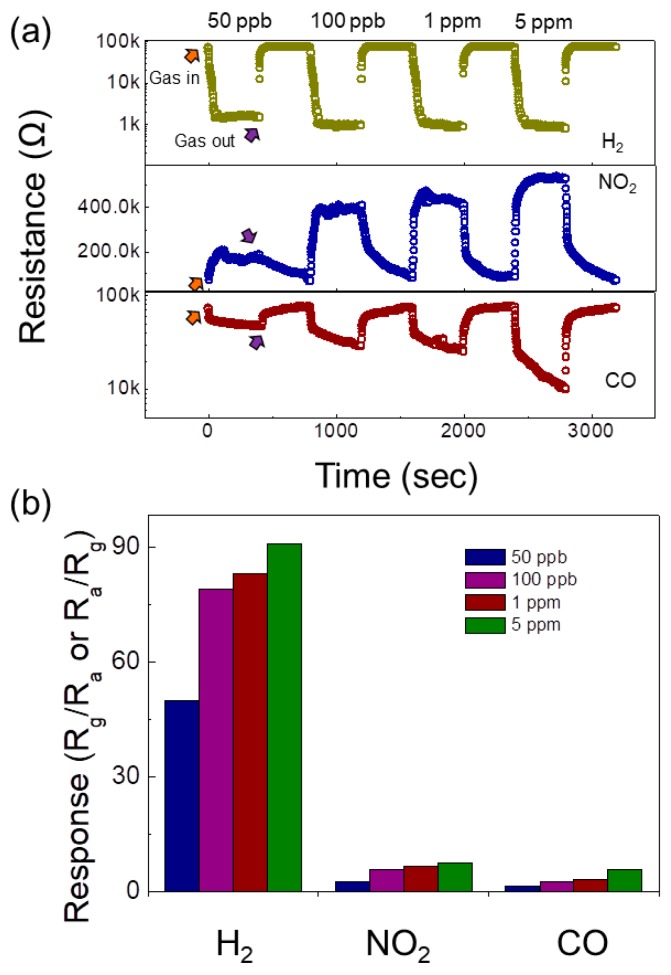
(**a**) Dynamic response curves of the 0.1 SnO_2_-loaded ZnO NFs gas sensor to H_2_, NO_2_ and CO gas at 300 °C. (**b**) Response histogram of the 0.1 SnO_2_-loaded ZnO NFs gas sensor at 300 °C.

The corresponding sensing transient curves at 300 °C were compared with that of hydrogen gas in Figure 7a. It should be noted that due to the oxidizing nature of NO_2_ gas, upon injection of NO_2_ gas, the resistance of the gas sensor increased, which was in contrast with the trend observed for reducing gases such as H_2_ and CO gases. Figure 7b shows the selectivity pattern of the 0.1 SnO_2_-loaded ZnO NFs gas sensor to H_2_, NO_2_ and CO gases. The response of the gas sensor to 50 ppb H_2_, NO_2_ and CO gases was 50, 2.62 and 1.57, respectively, which demonstrated the exceptionally high response of gas sensor to hydrogen gas. 

Table 1 [44,45,46,47,48,49,50,51,52,53,54] compares the response of some ZnO-based and SnO_2_-based gas sensors to hydrogen gas with the present 0.1 SnO_2_-loaded ZnO NFs gas sensor. Based on the data provided in this table, the present sensor had a much higher response to hydrogen gas than other reported hydrogen gas sensors and easily could detect ppb-levels of hydrogen gas.

**Figure 8:**
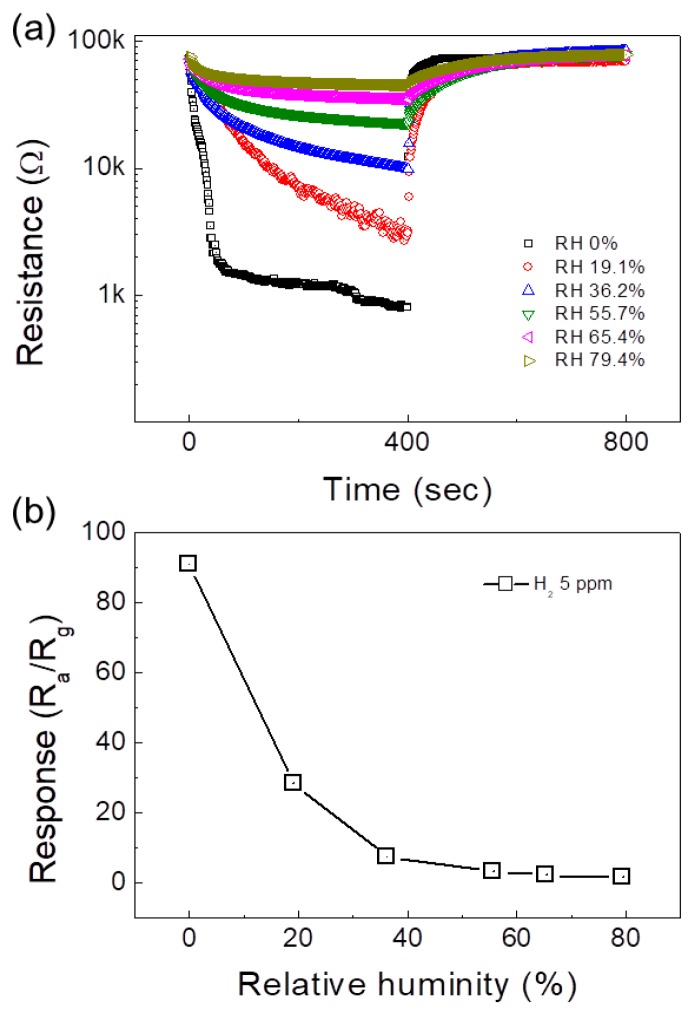
(**a**) Dynamic resistance curves of the 0.1 SnO_2_-loaded ZnO NF gas sensor to 5 ppm H_2_ gas in the presence of 0–79.4% relative humidity (RH). (**b**) Response to 5 ppm H_2_ versus RH%.

In real applications, there is always some humidity in the environment. Accordingly, we tested the response of the 0.1 SnO_2_-loaded ZnO NFs gas sensor to 5 ppm H_2_ gas in the presence of different levels of relative humidity (RH%) as shown in Figure 8a. As summarized in Figure 8b, with increasing of the RH%, the response decreases continuously. This was due to the fact that water molecules are likely to be adsorbed on the surfaces of the NFs and thus prevent the chemisorption of target gas species. Therefore, the number of the available sites for adsorption of H_2_ gas decreased, leading to a lower response of gas sensor in the presence of humidity.

**Figure 9:**
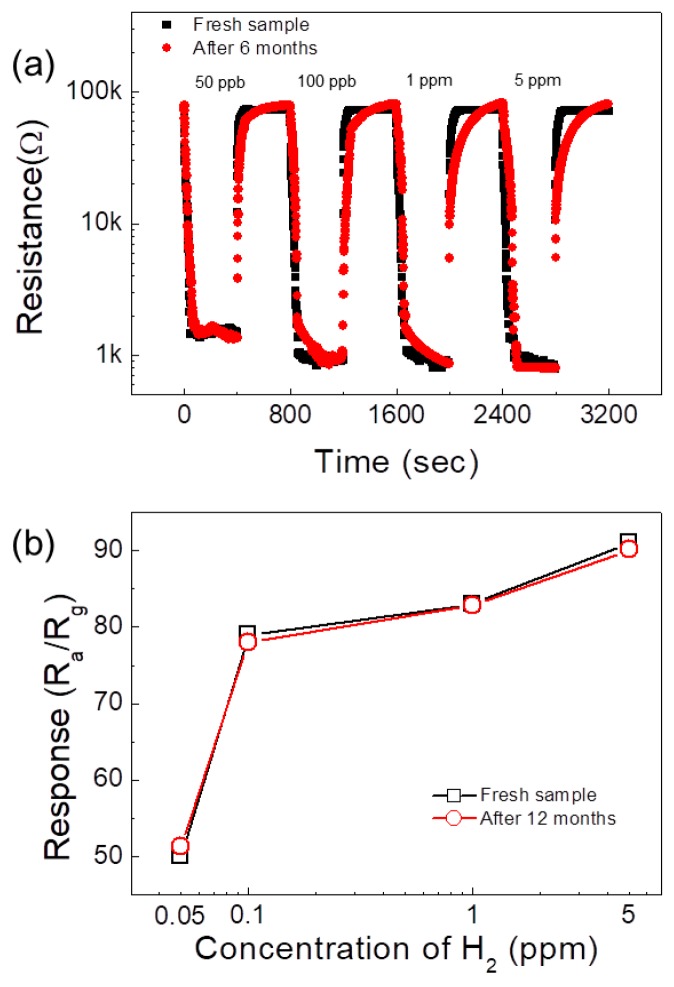
(**a**) Long-term stability curves of fresh and 6 months aged 0.1 SnO_2_-loaded ZnO NF gas sensor. (**b**) Response versus H_2_ gas concentration of fresh and 6 months aged 0.1 SnO_2_-loaded ZnO NF gas sensor.

Figure 9a,b shows the transient resistance curves and the calibration plots of a fresh and a six months aged 0.1 SnO_2_-loaded ZnO NFs sensor towards 50 ppb to 5 ppm of H_2_ gas, respectively. As can be seen, almost no drift was observed and the response of gas sensor, even after six months, was not changed. This demonstrated its high stability over time, which is important for long-term applications.

**Figure 10:**
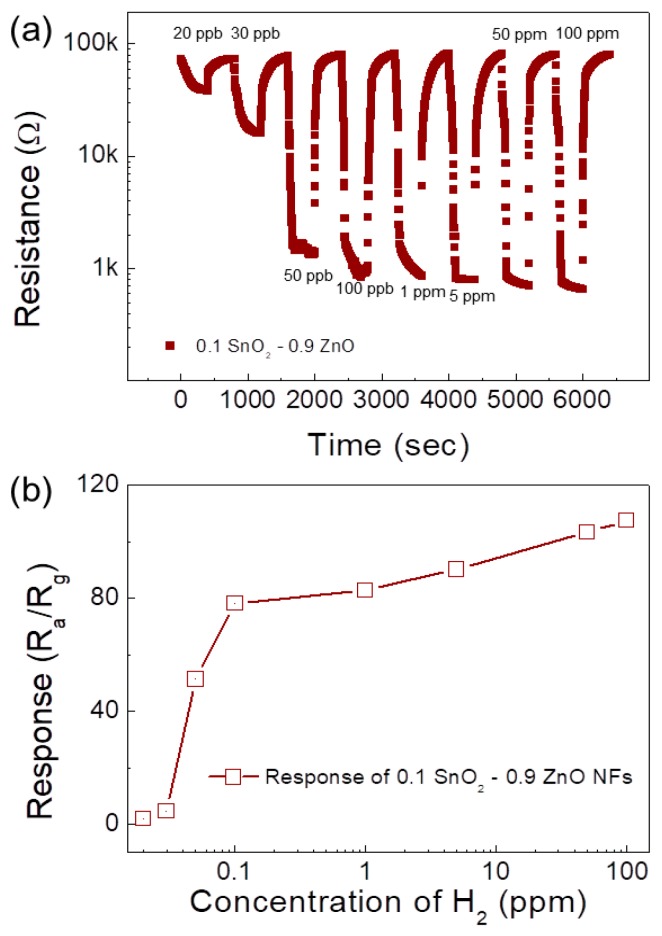
(**a**) Dynamic resistance curve of the 0.1 SnO_2_-loaded ZnO NF gas sensor towards 20 ppb to 100 ppm of H_2_ gas. (**b**) Response versus H_2_ gas concentration.

To explore the experimental detection limit of the 0.1 SnO_2_-loaded ZnO NFs gas sensor, it was exposed to various concentrations (20 ppb to 100 ppm) of H_2_ gas and the corresponding dynamic resistance curves are displayed in Figure 10a. The response is summarized in Figure 10b. As evidently shown, the sensor could even detect extremely low concentrations (20 ppb) of H_2_ gas. Also, the response of the gas sensor to 50 ppm and 100 ppm H_2_ gas were almost the same, which indicated saturation of gas sensor in this concentration was attained.

### 3.3. Sensing Mechanism

The sensing mechanism for resistive-based gas sensors is based on the variations of sensor’s resistance in the presence of target gases. Initially, in air, oxygen molecules will be adsorbed on the surface of gas sensor according to the following reactions [55]:

(1) $$O_{2}\left(g\right)\to O_{2}\left(ads\right)$$

(2) $$O_{2}+e^{-}\to O_{2}^{-}\left(ads\right)$$

(3) $$O_{2}^{-}\left(ads\right)+e^{-}\to 2O^{-}(ads)$$

(4) $$O^{-}\left(ads\right)+e^{-}\to O^{2-}\left(ads\right)$$

Due to adsorption of oxygen gas on the surface of gas sensor, electrons are extracted by adsorbed oxygen molecules and an ultra-thin, so-called electron-depletion layer (EDL), is formed on the surfaces of gas sensor. Therefore, for the SnO_2_-loaded ZnO gas sensors used in this study, EDLs will be formed on the bare surfaces of ZnO and SnO_2_, which are exposed to air. Upon injection of hydrogen gas, hydrogen can react with already adsorbed oxygen gas according to the following reaction:

(5) $$H_{2}+O^{-}\to H_{2}O+e$$

**Figure 11:**
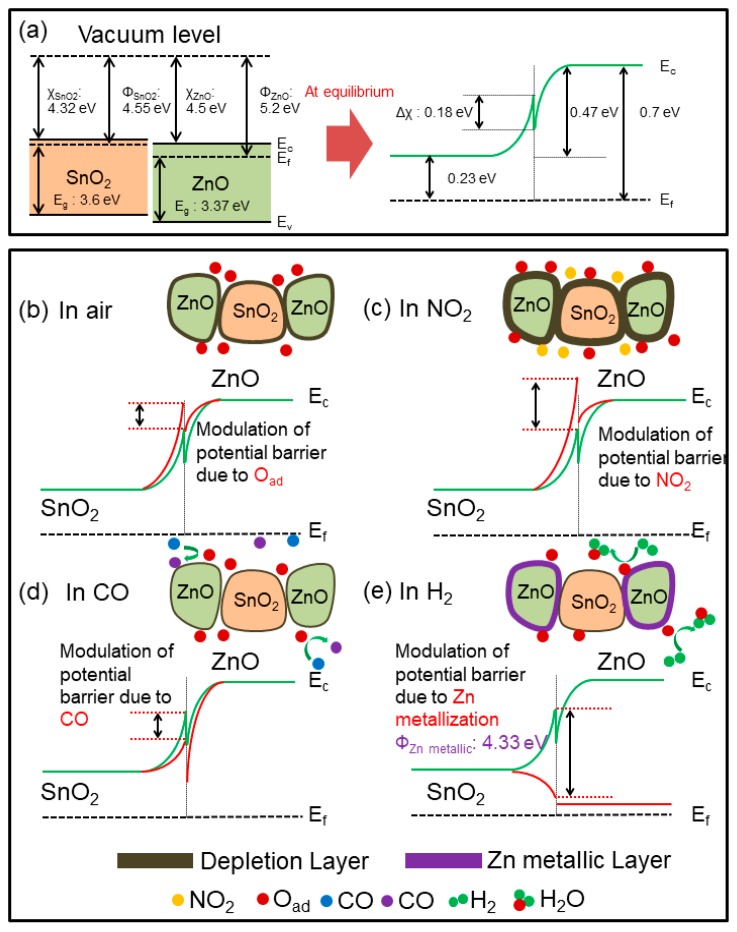
Schematics of sensing mechanism in the SnO_2_-loaded ZnO NFs gas sensor. (**a**) Energy-level diagram of ZnO-SnO_2_ in vacuum. The change of potential barriers in (**b**) air, (**c**) NO_2_, (**d**) CO, and (**e**) H_2_.

Thus, the generated electrons will come back to the surface of gas sensor and contract the width of EDLs, resulting in a significant decrease of the sensor’s resistance. Accordingly, a response due to variation of the width of the EDLs in exposed grains of ZnO and SnO_2_ will be result. As shown in Figure 2e, the NFs were composed of individual grains of ZnO and SnO_2_. Accordingly, in contact areas between ZnO-ZnO and SnO_2_-SnO_2_ homojunctions, potential barriers will be formed. Upon exposure to hydrogen, the height of the potential barrier will decrease, resulting in the facile transport of electrons and the decrease of the sensor’s resistance. Schematic illustrations showing the sensing mechanisms in SnO_2_-loaded ZnO NFs are presented in Figure 11.

Therefore, the second source of the resistance change was due to the presence of homojunctions. It should be noted that due to much higher amounts of ZnO relative to SnO_2_, the number of ZnO-ZnO homojunctions were significantly larger than that of the SnO_2_-SnO_2_ homojunctions. Another source of the resistance modulation came from the heterojunctions between ZnO and SnO_2_ grains. In SnO_2_-loaded ZnO NFs gas sensors, due to difference between the work functions of ZnO (Φ = 5.2 eV) [56] and SnO_2_ (Φ = 4.55 eV) [57], n-n heterojunctions could be formed in air. Accordingly, in intimate contact between ZnO and SnO_2_, electrons would be transferred from SnO_2_ to ZnO to equate the Fermi levels. Therefore, at the SnO_2_-ZnO heterointerfaces, electron accumulation layers and EDLs will be generated on the ZnO and SnO_2_ sides, respectively. As a result, band bending occurs and potential barriers will be formed in the interfaces between ZnO and SnO_2_, as shown in Figure 11a for a vacuum condition. In air, the height of the potential barrier increases relative to a vacuum, due to adsorption of oxygen gas (Figure 11b). In NO_2_ (oxidizing gas) and CO (reducing gas) environments, the height of potential barriers will increase and decrease, respectively (Figure 11c,d). However, in both cases the variations of the resistance modulations are not so significant and a low response would appear. 

After injection of hydrogen gas, due to the high reducing power of hydrogen gas and a relatively high sensing temperature, the H_2_ gas could reduce the surface of ZnO to metallic Zn with much lower resistivity than ZnO [48,49]. Without metallization effect, the variation of the potential barrier by the introduction of H_2_ gas would be almost similar to that of CO gas. Accordingly, upon exposure to H_2_ gas, ZnO would be converted to metallic Zn on the outer surfaces (Figure 11e) [43]. Accordingly, the ZnO-SnO_2_ heterojunctions were destroyed and electrons would be transferred from the metallic Zn (Φ = 4.33 eV) surface to ZnO. This semiconductor-to-metallic surface conversion in ZnO remarkably increased the modulation of resistance [58]. Upon injection of air, metallic Zn was converted back to ZnO, and its band structure would be recovered. 

The gas sensor with optimal composition of 0.1 SnO_2_-loaded ZnO exhibited the highest response to hydrogen gas. In fact, when the amount of SnO_2_ increased, the number of SnO_2_-ZnO heterojunctions increased, so a higher response was observed. However, further increases in the amount of SnO_2_ resulted in a decreased response of the gas sensor, due to possible agglomeration of SnO_2_ grains and increase of SnO_2_-SnO_2_ homojunctions instead of SnO_2_-ZnO heterojunctions. Further studies are needed to find the possible reasons for the decrease of sensing response at higher amounts of SnO_2_.

Overall the excellent selectivity of the optimized gas sensor could be attributed to (i) the metallization effect of ZnO in the presence of hydrogen gas, (ii) the smaller kinetic diameter (2.89 Å) [59] of H_2_ molecules relative to CO and NO_2_ molecules, and (iii) the optimal sensing temperature for enhanced adsorption and reaction of H_2_ with ZnO. In addition, the bond energy in H_2_ (436.0 KJ/mol) was much lower than that of CO (1076.5 KJ/mol) [60] and H_2_ has higher reactivity compared with CO, leading to higher response of the gas sensor to hydrogen gas, relative to CO gas. Other researchers have also reported a higher response of SnO_2_-ZnO gas sensors to hydrogen, relative to CO gas [56].

## 4. Conclusions

In brief, we synthesized xSnO_2_-loaded (x = 0.05, 0.1 and 0.15) ZnO NFs by a facile and low-cost electrospinning method, followed by high temperature calcination. FE-SEM and TEM characterization results demonstrated the formation of highly crystalline NFs with an approximate diameter of 100 nm with a desired chemical composition. The optimized gas sensor with composition of 0.1 SnO_2_-loaded ZnO NFs revealed the highest hydrogen response at an optimal temperature of 300 °C. The optimal gas sensor not only showed a high response to low concentrations of hydrogen gas, but also it showed an excellent selectivity to hydrogen gas. The gas sensing mechanism was related to the formation of SnO_2_-ZnO heterojunctions and the metallization effect of ZnO. The realized gas sensor in this study was able to detect ppb-levels of hydrogen in the atmosphere and could be used for practical applications.

## Figures and Tables

**Table 1:** Comparison between hydrogen gas sensing properties of the 0.1 SnO_2_-loaded ZnO NFs gas sensor with other ZnO-based or SnO_2_-based gas sensors reported in the literature.

Sensor	Conc. (ppm)	T (°C)	Response	Ref.
0.1 SnO_2_ loaded ZnO NFs	0.05	300	50.1 ^a^	Present work
WO_3_-ZnO	2000	200	13 ^a^	[44]
SnO_2_ NFs	1000	150	2.4 ^a^	[45]
ZnO Nanorods	100	340	5 ^a^	[46]
ZnO Nanorods	1000	250	11 ^a^	[47]
Mg doped ZnO thin films	5000	300	50 ^a^	[48]
Porous ZnO nanotubes	5000	200	8 ^a^	[49]
Ni-doped ZnO	10,000	150	43.4% ^b^	[50]
Pd-SnO_2_ composite microspheres	100	200	16.7 ^a^	[51]
Pd-SnO_2_ NFs	100	280	8.2 ^a^	[52]
Al-doped SnO_2_ NFs	100	340	7.7 ^a^	[53]
SnO_2_ nanosheets/carbon NFs	100	200	16.3 ^a^	[54]

Note: ^a^ = (R_a_/R_g_), ^b^ = (ΔR/R_a_).
